# (−)-Kolavenic acid

**DOI:** 10.1107/S1600536808014402

**Published:** 2008-05-17

**Authors:** Julio Zukerman-Schpector, Lucas Sousa Madureira, Gisele B. Messiano, Lucia M. X. Lopes, Edward R. T. Tiekink

**Affiliations:** a Department of Chemistry, Universidade Federal de São Carlos, 13565-905 São Carlos, SP, Brazil; b Chemistry Institute, São Paulo State University, UNESP, 14801-970, Araraquara, SP, Brazil; c Department of Chemistry, The University of Texas at San Antonio, One UTSA Circle, San Antonio, Texas 78249-0698, USA

## Abstract

In the two, almost identical, mol­ecules in the asymmetric unit of the title compound [systematic name: (*E*)-3-methyl-5-(1,2,4a,5-tetra­methyl-1,2,3,4,4a,7,8,8a-octa­hydro­naphthalen-1-yl)pent-2-enoic acid], C_20_H_32_O_2_, the rings are *trans* fused. The cyclo­hexane ring has a chair conformation and the cyclo­hexene ring a distorted half-boat conformation. The two independent mol­ecules are connected into a dimer *via* O—H⋯O hydrogen bonds. The dimers are associated into supra­molecular chains along *c via* C—H⋯O contacts.

## Related literature

For related structures, see: Puliti & Mattia (2000 ▶). For related literature, see: Lopes *et al.* (1987 ▶); Bomm *et al.* (1999 ▶); Messiano *et al.* (2008 ▶); Nascimento *et al.* (2004 ▶). For ring structure analysis, see: Cremer & Pople (1975 ▶); Spek (2003 ▶).
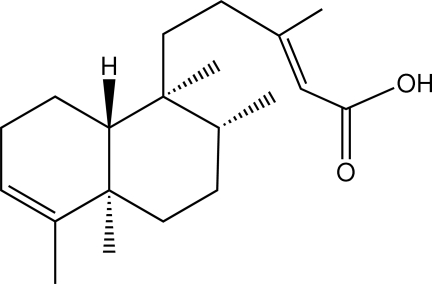


## Experimental

### 

#### Crystal data

C_20_H_32_O_2_
*M* *_r_* = 304.46
Orthorhombic, 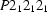

*a* = 12.5122 (3) Å
*b* = 15.5439 (4) Å
*c* = 19.1969 (4) Å
*V* = 3733.57 (15) Å^3^
*Z* = 8
Mo *K*α radiation
μ = 0.07 mm^−1^
*T* = 291 (2) K
0.42 × 0.20 × 0.18 mm

#### Data collection

Bruker APEXII CCD area-detector diffractometer
Absorption correction: none
48366 measured reflections
6240 independent reflections
3146 reflections with *I* > 2σ(*I*)
*R*_int_ = 0.052

#### Refinement

*R*[*F* ^2^ > 2σ(*F* ^2^)] = 0.050
*wR*(*F* ^2^) = 0.134
*S* = 1.00
6240 reflections
407 parameters
H-atom parameters constrained
Δρ_max_ = 0.11 e Å^−3^
Δρ_min_ = −0.12 e Å^−3^

### 

Data collection: *APEX2*, *COSMO* and *BIS* (Bruker, 2006 ▶); cell refinement: *SAINT* (Bruker, 2006 ▶); data reduction: *SAINT*; program(s) used to solve structure: *SIR97* (Altomare *et al.*, 1999 ▶); program(s) used to refine structure: *SHELXL97* (Sheldrick, 2008 ▶); molecular graphics: *DIAMOND* (Brandenburg, 2006 ▶); software used to prepare material for publication: *WinGX* (Farrugia, 1999 ▶).

## Supplementary Material

Crystal structure: contains datablocks global, I. DOI: 10.1107/S1600536808014402/ng2455sup1.cif

Structure factors: contains datablocks I. DOI: 10.1107/S1600536808014402/ng2455Isup2.hkl

Additional supplementary materials:  crystallographic information; 3D view; checkCIF report

## Figures and Tables

**Table 1:** Hydrogen-bond geometry (Å, °)

*D*—H⋯*A*	*D*—H	H⋯*A*	*D*⋯*A*	*D*—H⋯*A*
O101—H101⋯O202	0.82	1.82	2.625 (3)	168
O201—H201⋯O102	0.82	1.90	2.700 (2)	164
C212—H21*R*⋯O202^i^	0.96	2.60	3.519 (4)	159

Symmetry code: (i) 
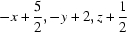
.

**Fig. 1.**
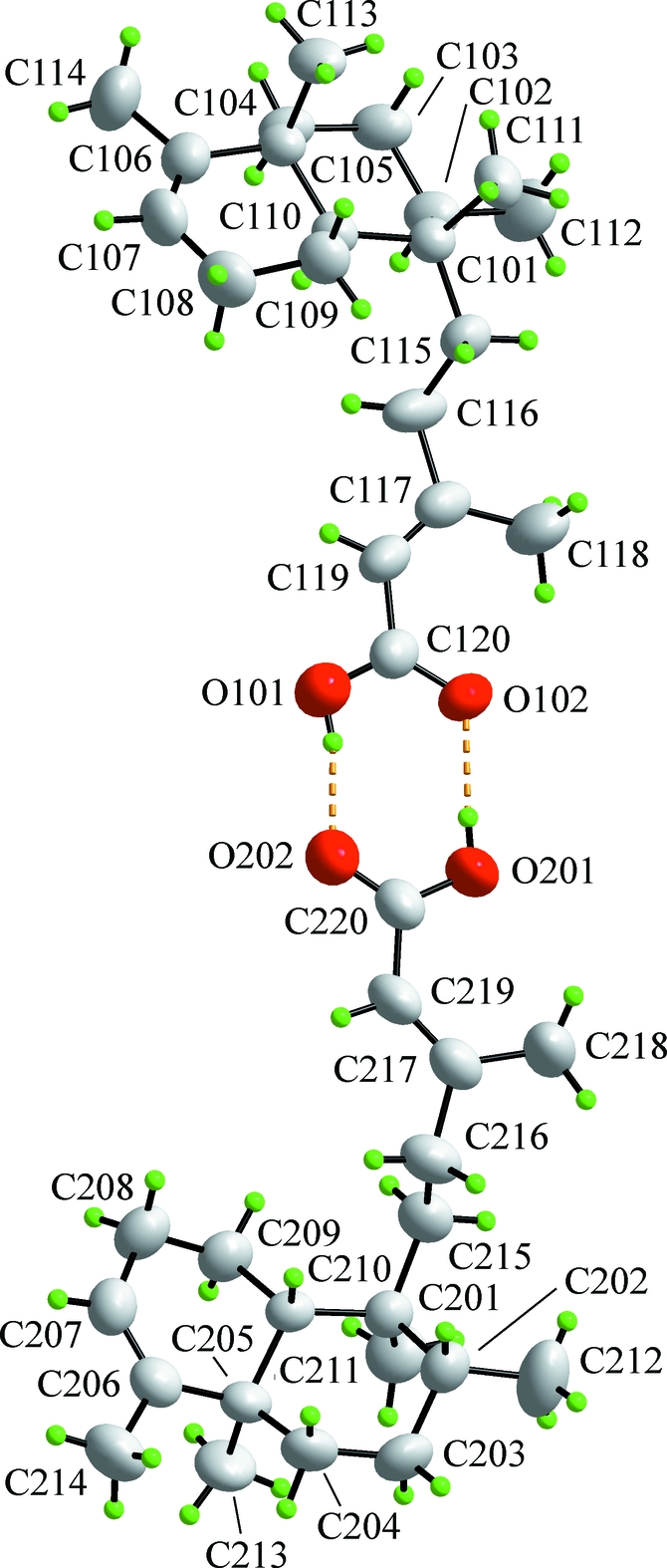
The molecular structures of the two independent molecules in (I) showing atom labelling scheme and displacement ellipsoids at the 35% probability level (arbitrary spheres for the H atoms).

**Fig. 2.**
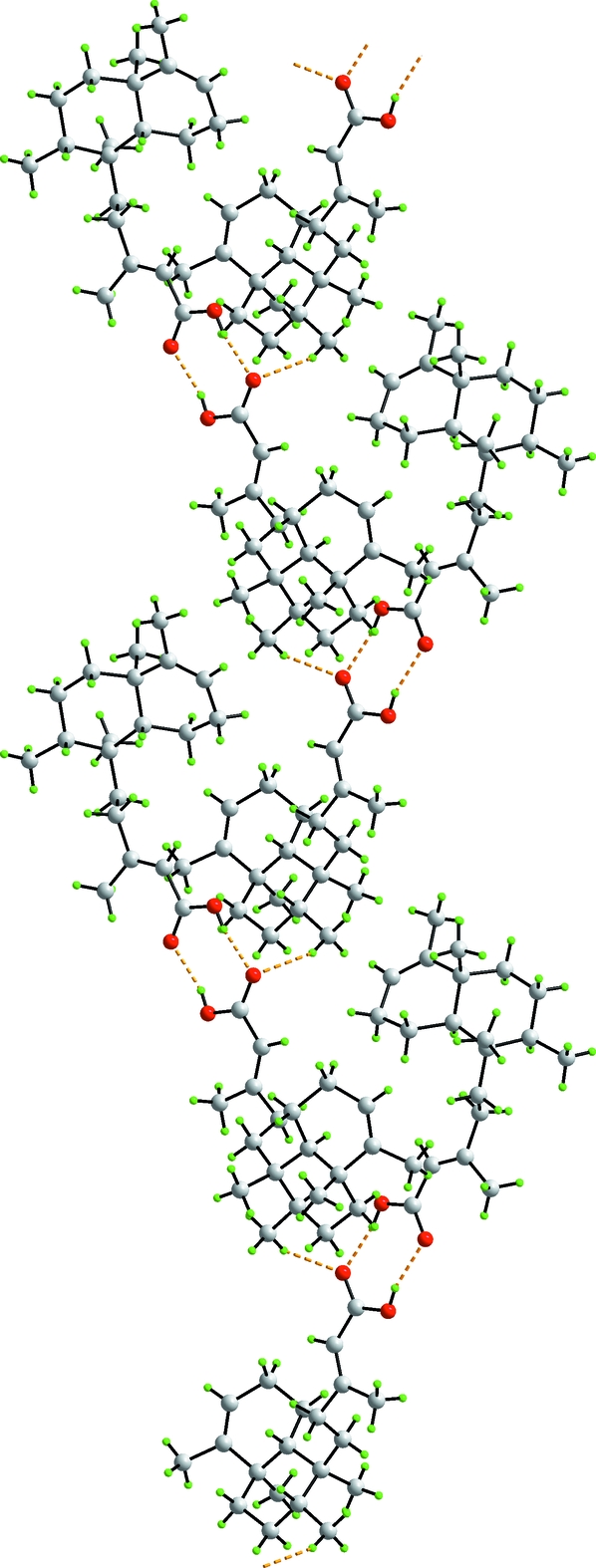
View of a supramolecular chain in (I) with O—H···O and C—H···O interactions shown as orange-dashed lines. Color code: O (red), N (blue), C (grey) & H (green).

## supplementary crystallographic information

### Comment

The title compound (I), Fig. 1, was studied as a part of an on-going screen of natural insecticides from *Aristolochia* species, which has become a promising route for the discovery of new compounds and/or botanical preparations which could be used in crop protection against *Anticarsia gemmatalis* H. (Lepidoptera: Noctuidae). Larvae of this insect represent the major defoliator pest of soybean crops in Brazil.

There are two almost identical independent molecules in the asymmetric unit, in fact superimposition of them, excluding H atoms, gives a rmsd of 0.016 Å (Spek, 2003). The major difference between the molecules is manifested in the relative orientations of the carboxylic acid residues so that in one molecule the carbonyl-O102 atom is *syn* to the methyl-C118 group whereas the opposite is true for the second independent molecule. In each molecule the rings are *trans* fused and the cyclohexane ring is in an almost undistorted chair conformation. The cyclohexene ring is in a distorted half-boat conformation, the ring-puckering parameters (Cremer & Pople, 1975) are q_2_ = 0.400 (3) Å (0.406 (3) Å for the second molecule), q_3_ = -0.341 (3) Å (-0.336 (3) Å), Q = 0.525 (2) ° (0.527 (2) °), and φ_2_ = 106.6 (4)° (107.3 (4) °). The absolute configuration was established based on the [α_D_] = -41.1° (c 1.0, CHCl_3_) and the results reported in Bomm *et al.* (1999).

The independent molecules in (I) are connected *via* cooperative O—H···O contacts that form the eight-membered {···H—O—C=O}_2_ synthon, Table 1. The resultant dimeric aggregates are linked into a supramolecular chain along the c-direction *via* C212—H21R···O202 contacts, Fig. 2.

### Experimental

Compound (I), (-)-kolavenic acid, was obtained from the hexane extract of the roots of *Aristolochia malmeana* Hoehne (Aristolochiaceae). Colorless crystals were obtained from the slow evaporation of a MeOH solution of (I) held at 283 K; m.p. 370–371 K. NMR (CDCl_3_, p.p.m.): δ 0.93 (1*H*, t, w_1/2_ = 7.0 Hz, H-1_a_), 1.36 (1*H*, m, H-1_b_), 1.99 (1*H*, m, H-2_a_), 1.94 (1*H*, m, H-2_b_), 5.13 (1*H*, br s, H-3), 1.66 (1*H*, dt, *J* = 13.0, 3.0 Hz, H-6_a_), 1.12 (1*H*, ddd, *J* = 13.0, 12.0, 4.2 Hz, H-6_b_), 1.33 (1*H*, m, H-7a), 1.39 (1*H*, m, H-7_b_), 1.37 (1*H*, m, H-8), 1.27 (1*H*, dd, *J* = 12.0, 1.5 Hz, H-10), 1.34 (1*H*, ddd, *J* = 14.0, 13.0, 4.5 Hz, H-11_a_), 1.48 (1*H*, ddd, *J* = 14.0, 12.5, 5.0 Hz, H-11_b_), 1.97 (1*H*, td, *J* = 13.0, 4.5 Hz, H-12_a_), 1.90 (1*H*, ddd, *J* = 13.0, 12.5, 5.0 Hz, H-12_b_), 5.62 (1*H*, dq, *J* = 2.5, 1.0 Hz, H-14), 2.11 (3*H*, br d, *J* = 1.0 Hz, H-16), 0.76 (3*H*, d, *J* = 6.0 Hz, H-17), 1.53 (3*H*, br s, H-18), 0.94 (3*H*, s, H-19), 0.68 (3*H*, s, H-20). [α_D_] = -41.1° in agreement with Bomm *et al.* (1999) and Messiano *et al.* (2008).

### Refinement

In the absence of significant anomalous scattering effects, Friedel pairs were averaged in the final refinement. The H atoms were refined in the riding-model approximation with C—H = 0.93 - 0.98 Å and (0.82 for O—H), and with *U*_iso_(H) = 1.5*U*_eq_(methyl-C) or 1.2*U*_eq_(remaining-C and –O).

### Crystal data

**Table:**

C_20_H_32_O_2_	*F*_000_ = 1344
*M**_r_* = 304.46	*D*_x_ = 1.083 Mg m^−^^3^
Orthorhombic, *P*2_1_2_1_2_1_	Mo *K*α radiation λ = 0.71073 Å
Hall symbol: P 2ac 2ab	Cell parameters from 9536 reflections
*a* = 12.5122 (3) Å	θ = 2.3–21.8º
*b* = 15.5439 (4) Å	µ = 0.07 mm^−^^1^
*c* = 19.1969 (4) Å	*T* = 291 (2) K
*V* = 3733.57 (15) Å^3^	Irregular, colourless
*Z* = 8	0.42 × 0.20 × 0.18 mm

### Data collection

**Table:**

Bruker APEXII CCD area-detector diffractometer	3146 reflections with *I* > 2σ(*I*)
Radiation source: fine-focus sealed tube	*R*_int_ = 0.052
Monochromator: graphite	θ_max_ = 30.5º
*T* = 291(2) K	θ_min_ = 2.7º
φ and ω scans	*h* = −17→17
Absorption correction: none	*k* = −22→21
48366 measured reflections	*l* = −27→23
6240 independent reflections	

### Refinement

**Table:**

Refinement on *F*^2^	Hydrogen site location: inferred from neighbouring sites
Least-squares matrix: full	H-atom parameters constrained
*R*[*F*^2^ > 2σ(*F*^2^)] = 0.050	*w* = 1/[σ^2^(*F*_o_^2^) + (0.0656*P*)^2^] where *P* = (*F*_o_^2^ + 2*F*_c_^2^)/3
*wR*(*F*^2^) = 0.134	(Δ/σ)_max_ < 0.001
*S* = 1.00	Δρ_max_ = 0.11 e Å^−^^3^
6240 reflections	Δρ_min_ = −0.12 e Å^−^^3^
407 parameters	Extinction correction: none
Primary atom site location: structure-invariant direct methods	Absolute structure: Friedel pairs were merged
Secondary atom site location: difference Fourier map	

### Special details

**Table:**

Geometry. All e.s.d.'s (except the e.s.d. in the dihedral angle between two l.s. planes) are estimated using the full covariance matrix. The cell e.s.d.'s are taken into account individually in the estimation of e.s.d.'s in distances, angles and torsion angles; correlations between e.s.d.'s in cell parameters are only used when they are defined by crystal symmetry. An approximate (isotropic) treatment of cell e.s.d.'s is used for estimating e.s.d.'s involving l.s. planes.
Refinement. Refinement of *F*^2^ against ALL reflections. The weighted *R*-factor *wR* and goodness of fit *S* are based on *F*^2^, conventional *R*-factors *R* are based on *F*, with *F* set to zero for negative *F*^2^. The threshold expression of *F*^2^ > σ(*F*^2^) is used only for calculating *R*-factors(gt) *etc*. and is not relevant to the choice of reflections for refinement. *R*-factors based on *F*^2^ are statistically about twice as large as those based on *F*, and *R*- factors based on ALL data will be even larger.

### Fractional atomic coordinates and isotropic or equivalent isotropic displacement parameters (Å^2^)

**Table:**

	*x*	*y*	*z*	*U*_iso_*/*U*_eq_	
O101	1.0346 (2)	1.00268 (16)	0.40732 (10)	0.0971 (7)	
H101	1.0583	1.0109	0.4466	0.117*	
O102	0.92225 (17)	0.92145 (12)	0.46736 (8)	0.0785 (5)	
C101	0.76320 (18)	0.77650 (14)	0.14588 (11)	0.0507 (5)	
C102	0.65232 (18)	0.81476 (17)	0.12872 (11)	0.0595 (6)	
H102	0.6514	0.8730	0.1484	0.071*	
C103	0.63223 (19)	0.82452 (18)	0.05042 (12)	0.0629 (6)	
H10C	0.6296	0.7679	0.0293	0.076*	
H10D	0.5633	0.8517	0.0433	0.076*	
C104	0.71754 (19)	0.87743 (17)	0.01459 (11)	0.0598 (6)	
H10A	0.7174	0.9351	0.0338	0.072*	
H10B	0.7009	0.8817	−0.0346	0.072*	
C105	0.83006 (17)	0.83772 (14)	0.02341 (10)	0.0517 (5)	
C106	0.9156 (2)	0.89798 (18)	−0.00476 (12)	0.0668 (7)	
C107	1.0092 (2)	0.90785 (19)	0.02659 (14)	0.0747 (8)	
H107	1.0581	0.9448	0.0056	0.090*	
C108	1.0430 (2)	0.86495 (19)	0.09252 (15)	0.0764 (8)	
H10G	1.0494	0.9079	0.1290	0.092*	
H10H	1.1128	0.8391	0.0858	0.092*	
C109	0.96454 (18)	0.79603 (17)	0.11559 (13)	0.0628 (6)	
H10E	0.9750	0.7837	0.1646	0.075*	
H10F	0.9774	0.7436	0.0896	0.075*	
C110	0.85005 (16)	0.82610 (14)	0.10350 (10)	0.0473 (5)	
H110	0.8482	0.8847	0.1223	0.057*	
C111	0.7674 (2)	0.67820 (15)	0.13196 (13)	0.0672 (7)	
H11E	0.7390	0.6663	0.0865	0.101*	
H11F	0.7256	0.6487	0.1665	0.101*	
H11G	0.8401	0.6588	0.1343	0.101*	
C112	0.5582 (2)	0.7661 (2)	0.16212 (15)	0.0902 (9)	
H11N	0.5548	0.7089	0.1434	0.135*	
H11O	0.4927	0.7958	0.1521	0.135*	
H11P	0.5684	0.7632	0.2116	0.135*	
C113	0.8360 (2)	0.75442 (18)	−0.01978 (12)	0.0678 (7)	
H11H	0.9004	0.7239	−0.0084	0.102*	
H11I	0.8360	0.7685	−0.0685	0.102*	
H11J	0.7753	0.7189	−0.0094	0.102*	
C114	0.8941 (3)	0.9435 (2)	−0.07248 (15)	0.1023 (11)	
H11Q	0.9553	0.9772	−0.0852	0.153*	
H11R	0.8331	0.9804	−0.0673	0.153*	
H11S	0.8801	0.9018	−0.1082	0.153*	
C115	0.7858 (2)	0.78741 (15)	0.22501 (11)	0.0609 (6)	
H11A	0.7346	0.7525	0.2504	0.073*	
H11B	0.8562	0.7642	0.2346	0.073*	
C116	0.7812 (3)	0.87882 (18)	0.25429 (12)	0.0834 (9)	
H11C	0.8247	0.9161	0.2253	0.100*	
H11D	0.7081	0.8994	0.2519	0.100*	
C117	0.8197 (3)	0.88539 (18)	0.32881 (11)	0.0705 (7)	
C118	0.7538 (3)	0.8379 (2)	0.38179 (14)	0.0987 (10)	
H11K	0.7705	0.8588	0.4276	0.148*	
H11L	0.7695	0.7775	0.3792	0.148*	
H11M	0.6793	0.8470	0.3724	0.148*	
C119	0.9048 (3)	0.93132 (18)	0.34285 (12)	0.0740 (8)	
H119	0.9387	0.9551	0.3043	0.089*	
C120	0.9549 (2)	0.95094 (17)	0.41065 (12)	0.0661 (7)	
O201	1.01800 (18)	0.96623 (14)	0.58806 (10)	0.0910 (6)	
H201	1.0001	0.9486	0.5495	0.109*	
O202	1.13450 (18)	1.03856 (14)	0.52400 (10)	0.0910 (6)	
C201	1.19128 (18)	1.20921 (15)	0.85454 (11)	0.0546 (6)	
C202	1.2484 (2)	1.15676 (16)	0.91200 (12)	0.0658 (7)	
H202	1.2947	1.1154	0.8880	0.079*	
C203	1.3211 (2)	1.21238 (19)	0.95685 (12)	0.0782 (8)	
H20E	1.2778	1.2535	0.9823	0.094*	
H20F	1.3571	1.1761	0.9906	0.094*	
C204	1.4038 (2)	1.26039 (17)	0.91493 (12)	0.0706 (7)	
H20A	1.4509	1.2192	0.8926	0.085*	
H20B	1.4468	1.2952	0.9462	0.085*	
C205	1.3545 (2)	1.31906 (15)	0.85873 (10)	0.0560 (6)	
C206	1.4416 (2)	1.35422 (15)	0.81050 (13)	0.0636 (6)	
C207	1.4261 (2)	1.36223 (17)	0.74262 (13)	0.0719 (7)	
H207	1.4824	1.3844	0.7165	0.086*	
C208	1.3270 (2)	1.33897 (19)	0.70427 (12)	0.0768 (8)	
H20G	1.3420	1.2908	0.6737	0.092*	
H20H	1.3054	1.3872	0.6755	0.092*	
C209	1.2348 (2)	1.31491 (18)	0.75260 (12)	0.0658 (6)	
H20C	1.1824	1.2814	0.7272	0.079*	
H20D	1.2002	1.3667	0.7695	0.079*	
C210	1.27730 (17)	1.26243 (14)	0.81438 (10)	0.0496 (5)	
H210	1.3237	1.2190	0.7931	0.060*	
C211	1.1011 (2)	1.2658 (2)	0.88417 (15)	0.0793 (8)	
H21H	1.0777	1.3057	0.8492	0.119*	
H21I	1.1270	1.2969	0.9239	0.119*	
H21J	1.0422	1.2300	0.8979	0.119*	
C212	1.1724 (3)	1.1040 (3)	0.95738 (18)	0.1120 (12)	
H21Q	1.1314	1.1420	0.9864	0.168*	
H21R	1.2129	1.0655	0.9861	0.168*	
H21S	1.1250	1.0715	0.9281	0.168*	
C213	1.3019 (3)	1.39861 (18)	0.89400 (14)	0.0816 (8)	
H21E	1.3566	1.4348	0.9133	0.122*	
H21F	1.2550	1.3797	0.9305	0.122*	
H21G	1.2619	1.4304	0.8600	0.122*	
C214	1.5467 (3)	1.3827 (2)	0.84228 (17)	0.0938 (10)	
H21N	1.5892	1.4108	0.8075	0.141*	
H21O	1.5844	1.3333	0.8596	0.141*	
H21P	1.5330	1.4218	0.8799	0.141*	
C215	1.1350 (2)	1.14633 (17)	0.80335 (13)	0.0666 (7)	
H21A	1.0793	1.1168	0.8291	0.080*	
H21B	1.0998	1.1808	0.7680	0.080*	
C216	1.2006 (2)	1.07826 (18)	0.76594 (14)	0.0746 (7)	
H21C	1.2620	1.1052	0.7441	0.089*	
H21D	1.2267	1.0367	0.7996	0.089*	
C217	1.1357 (2)	1.03233 (16)	0.71123 (14)	0.0678 (7)	
C218	1.0479 (3)	0.9751 (2)	0.73784 (15)	0.1064 (12)	
H21K	1.0457	0.9233	0.7107	0.160*	
H21L	0.9807	1.0045	0.7341	0.160*	
H21M	1.0613	0.9610	0.7857	0.160*	
C219	1.1563 (2)	1.04597 (17)	0.64412 (14)	0.0721 (7)	
H219	1.2155	1.0805	0.6353	0.087*	
C220	1.1003 (2)	1.01506 (17)	0.58192 (14)	0.0679 (7)	

### Atomic displacement parameters (Å^2^)

**Table:**

	*U*^11^	*U*^22^	*U*^33^	*U*^12^	*U*^13^	*U*^23^
O101	0.1228 (18)	0.1026 (16)	0.0661 (11)	−0.0288 (16)	−0.0021 (12)	0.0042 (11)
O102	0.1089 (14)	0.0789 (12)	0.0478 (8)	−0.0094 (11)	−0.0006 (9)	0.0002 (8)
C101	0.0526 (13)	0.0479 (13)	0.0516 (11)	0.0024 (11)	0.0030 (9)	−0.0041 (10)
C102	0.0501 (13)	0.0707 (16)	0.0576 (12)	0.0062 (13)	0.0043 (10)	−0.0132 (11)
C103	0.0501 (13)	0.0779 (17)	0.0608 (12)	0.0097 (13)	−0.0028 (10)	−0.0154 (12)
C104	0.0632 (15)	0.0679 (16)	0.0484 (11)	0.0089 (13)	−0.0080 (10)	−0.0058 (11)
C105	0.0519 (13)	0.0542 (14)	0.0489 (10)	−0.0026 (11)	0.0019 (9)	−0.0062 (10)
C106	0.0736 (18)	0.0674 (17)	0.0595 (13)	−0.0055 (14)	0.0091 (12)	−0.0003 (11)
C107	0.0674 (18)	0.0759 (19)	0.0808 (17)	−0.0213 (15)	0.0112 (14)	0.0013 (15)
C108	0.0547 (15)	0.087 (2)	0.0875 (18)	−0.0099 (15)	−0.0005 (13)	−0.0076 (15)
C109	0.0517 (14)	0.0677 (17)	0.0690 (13)	0.0005 (13)	−0.0041 (11)	0.0012 (12)
C110	0.0466 (12)	0.0445 (12)	0.0508 (10)	0.0026 (10)	−0.0005 (9)	−0.0047 (9)
C111	0.0730 (17)	0.0517 (15)	0.0769 (15)	−0.0064 (13)	0.0081 (13)	−0.0040 (12)
C112	0.0582 (16)	0.130 (3)	0.0824 (18)	−0.0045 (18)	0.0165 (14)	−0.0002 (18)
C113	0.0678 (16)	0.0763 (17)	0.0595 (13)	−0.0024 (14)	0.0094 (12)	−0.0217 (12)
C114	0.116 (3)	0.114 (3)	0.0773 (18)	−0.026 (2)	0.0037 (17)	0.0291 (18)
C115	0.0730 (16)	0.0609 (15)	0.0486 (11)	0.0045 (13)	−0.0012 (10)	0.0023 (10)
C116	0.134 (3)	0.0673 (17)	0.0486 (12)	0.0166 (18)	−0.0167 (14)	−0.0087 (11)
C117	0.103 (2)	0.0588 (16)	0.0491 (12)	0.0123 (17)	−0.0046 (13)	−0.0020 (11)
C118	0.115 (2)	0.126 (3)	0.0552 (14)	−0.016 (2)	0.0099 (15)	−0.0095 (16)
C119	0.117 (2)	0.0600 (16)	0.0451 (11)	0.0014 (18)	0.0042 (13)	0.0016 (11)
C120	0.092 (2)	0.0499 (14)	0.0563 (13)	0.0022 (15)	0.0011 (13)	0.0026 (11)
O201	0.1098 (16)	0.0939 (15)	0.0694 (11)	−0.0434 (13)	−0.0103 (11)	−0.0026 (11)
O202	0.1104 (16)	0.0877 (14)	0.0749 (11)	−0.0261 (13)	0.0126 (11)	−0.0057 (10)
C201	0.0578 (13)	0.0531 (14)	0.0528 (11)	0.0057 (12)	−0.0021 (10)	0.0010 (10)
C202	0.0770 (17)	0.0646 (16)	0.0557 (12)	−0.0001 (14)	−0.0019 (12)	0.0094 (11)
C203	0.101 (2)	0.088 (2)	0.0464 (11)	0.0010 (17)	−0.0109 (13)	0.0033 (12)
C204	0.0850 (17)	0.0684 (16)	0.0584 (12)	−0.0051 (15)	−0.0223 (13)	−0.0048 (12)
C205	0.0695 (15)	0.0471 (13)	0.0513 (10)	0.0029 (12)	−0.0058 (11)	−0.0102 (9)
C206	0.0709 (16)	0.0483 (14)	0.0717 (15)	0.0015 (13)	−0.0016 (12)	−0.0070 (11)
C207	0.0831 (19)	0.0599 (16)	0.0726 (16)	−0.0003 (14)	0.0096 (14)	0.0064 (12)
C208	0.088 (2)	0.086 (2)	0.0563 (13)	0.0049 (17)	−0.0013 (13)	0.0107 (13)
C209	0.0720 (16)	0.0679 (16)	0.0577 (13)	0.0086 (14)	−0.0099 (11)	0.0063 (12)
C210	0.0562 (13)	0.0474 (13)	0.0452 (10)	0.0077 (11)	−0.0029 (9)	−0.0053 (9)
C211	0.0693 (16)	0.084 (2)	0.0841 (16)	0.0134 (16)	0.0101 (14)	−0.0062 (15)
C212	0.103 (2)	0.129 (3)	0.105 (2)	−0.019 (2)	0.0017 (19)	0.051 (2)
C213	0.104 (2)	0.0619 (16)	0.0795 (16)	0.0050 (16)	0.0102 (16)	−0.0254 (13)
C214	0.091 (2)	0.085 (2)	0.105 (2)	−0.0210 (19)	−0.0075 (18)	−0.0182 (18)
C215	0.0614 (15)	0.0675 (16)	0.0710 (14)	−0.0013 (14)	−0.0095 (12)	−0.0035 (12)
C216	0.0775 (18)	0.0654 (17)	0.0807 (16)	−0.0008 (15)	−0.0161 (14)	−0.0152 (13)
C217	0.0700 (16)	0.0535 (15)	0.0798 (16)	−0.0062 (14)	−0.0103 (13)	−0.0049 (12)
C218	0.141 (3)	0.101 (2)	0.0775 (18)	−0.054 (2)	−0.020 (2)	0.0130 (17)
C219	0.0713 (17)	0.0640 (17)	0.0810 (16)	−0.0178 (14)	0.0026 (14)	−0.0170 (13)
C220	0.0780 (18)	0.0523 (15)	0.0736 (16)	−0.0091 (14)	0.0035 (14)	−0.0079 (12)

### Geometric parameters (Å, °)

**Table:**

O101—C120	1.283 (3)	O201—C220	1.285 (3)
O101—H101	0.8200	O201—H201	0.8200
O102—C120	1.250 (3)	O202—C220	1.246 (3)
C101—C102	1.545 (3)	C201—C211	1.540 (3)
C101—C111	1.552 (3)	C201—C202	1.547 (3)
C101—C115	1.554 (3)	C201—C215	1.555 (3)
C101—C110	1.561 (3)	C201—C210	1.561 (3)
C102—C103	1.532 (3)	C202—C203	1.522 (4)
C102—C112	1.540 (4)	C202—C212	1.528 (4)
C102—H102	0.9800	C202—H202	0.9800
C103—C104	1.513 (4)	C203—C204	1.509 (4)
C103—H10C	0.9700	C203—H20E	0.9700
C103—H10D	0.9700	C203—H20F	0.9700
C104—C105	1.546 (3)	C204—C205	1.542 (3)
C104—H10A	0.9700	C204—H20A	0.9700
C104—H10B	0.9700	C204—H20B	0.9700
C105—C106	1.522 (4)	C205—C206	1.531 (3)
C105—C113	1.539 (3)	C205—C213	1.555 (3)
C105—C110	1.568 (3)	C205—C210	1.560 (3)
C106—C107	1.325 (4)	C206—C207	1.323 (3)
C106—C114	1.504 (4)	C206—C214	1.516 (4)
C107—C108	1.492 (4)	C207—C208	1.487 (4)
C107—H107	0.9300	C207—H207	0.9300
C108—C109	1.519 (4)	C208—C209	1.526 (4)
C108—H10G	0.9700	C208—H20G	0.9700
C108—H10H	0.9700	C208—H20H	0.9700
C109—C110	1.525 (3)	C209—C210	1.534 (3)
C109—H10E	0.9700	C209—H20C	0.9700
C109—H10F	0.9700	C209—H20D	0.9700
C110—H110	0.9800	C210—H210	0.9800
C111—H11E	0.9600	C211—H21H	0.9600
C111—H11F	0.9600	C211—H21I	0.9600
C111—H11G	0.9600	C211—H21J	0.9600
C112—H11N	0.9600	C212—H21Q	0.9600
C112—H11O	0.9600	C212—H21R	0.9600
C112—H11P	0.9600	C212—H21S	0.9600
C113—H11H	0.9600	C213—H21E	0.9600
C113—H11I	0.9600	C213—H21F	0.9600
C113—H11J	0.9600	C213—H21G	0.9600
C114—H11Q	0.9600	C214—H21N	0.9600
C114—H11R	0.9600	C214—H21O	0.9600
C114—H11S	0.9600	C214—H21P	0.9600
C115—C116	1.529 (4)	C215—C216	1.519 (3)
C115—H11A	0.9700	C215—H21A	0.9700
C115—H11B	0.9700	C215—H21B	0.9700
C116—C117	1.513 (3)	C216—C217	1.507 (3)
C116—H11C	0.9700	C216—H21C	0.9700
C116—H11D	0.9700	C216—H21D	0.9700
C117—C119	1.310 (4)	C217—C219	1.331 (4)
C117—C118	1.503 (4)	C217—C218	1.503 (4)
C118—H11K	0.9600	C218—H21K	0.9600
C118—H11L	0.9600	C218—H21L	0.9600
C118—H11M	0.9600	C218—H21M	0.9600
C119—C120	1.476 (4)	C219—C220	1.465 (4)
C119—H119	0.9300	C219—H219	0.9300
			
C120—O101—H101	109.5	C220—O201—H201	109.5
C102—C101—C111	111.9 (2)	C211—C201—C202	112.09 (19)
C102—C101—C115	109.25 (18)	C211—C201—C215	105.1 (2)
C111—C101—C115	105.63 (19)	C202—C201—C215	109.19 (19)
C102—C101—C110	108.90 (17)	C211—C201—C210	112.66 (19)
C111—C101—C110	111.90 (18)	C202—C201—C210	108.25 (18)
C115—C101—C110	109.20 (18)	C215—C201—C210	109.47 (18)
C103—C102—C112	109.4 (2)	C203—C202—C212	110.8 (2)
C103—C102—C101	113.24 (18)	C203—C202—C201	112.3 (2)
C112—C102—C101	114.1 (2)	C212—C202—C201	113.7 (2)
C103—C102—H102	106.5	C203—C202—H202	106.5
C112—C102—H102	106.5	C212—C202—H202	106.5
C101—C102—H102	106.5	C201—C202—H202	106.5
C104—C103—C102	112.6 (2)	C204—C203—C202	112.92 (19)
C104—C103—H10C	109.1	C204—C203—H20E	109.0
C102—C103—H10C	109.1	C202—C203—H20E	109.0
C104—C103—H10D	109.1	C204—C203—H20F	109.0
C102—C103—H10D	109.1	C202—C203—H20F	109.0
H10C—C103—H10D	107.8	H20E—C203—H20F	107.8
C103—C104—C105	112.06 (19)	C203—C204—C205	113.0 (2)
C103—C104—H10A	109.2	C203—C204—H20A	109.0
C105—C104—H10A	109.2	C205—C204—H20A	109.0
C103—C104—H10B	109.2	C203—C204—H20B	109.0
C105—C104—H10B	109.2	C205—C204—H20B	109.0
H10A—C104—H10B	107.9	H20A—C204—H20B	107.8
C106—C105—C113	107.00 (18)	C206—C205—C204	110.4 (2)
C106—C105—C104	110.85 (19)	C206—C205—C213	106.3 (2)
C113—C105—C104	108.70 (18)	C204—C205—C213	109.56 (19)
C106—C105—C110	107.88 (18)	C206—C205—C210	108.19 (17)
C113—C105—C110	115.05 (19)	C204—C205—C210	107.22 (19)
C104—C105—C110	107.37 (16)	C213—C205—C210	115.1 (2)
C107—C106—C114	119.8 (3)	C207—C206—C214	119.7 (3)
C107—C106—C105	122.1 (2)	C207—C206—C205	121.7 (2)
C114—C106—C105	118.1 (2)	C214—C206—C205	118.6 (2)
C106—C107—C108	125.7 (2)	C206—C207—C208	125.9 (3)
C106—C107—H107	117.1	C206—C207—H207	117.0
C108—C107—H107	117.1	C208—C207—H207	117.0
C107—C108—C109	112.3 (2)	C207—C208—C209	112.9 (2)
C107—C108—H10G	109.2	C207—C208—H20G	109.0
C109—C108—H10G	109.2	C209—C208—H20G	109.0
C107—C108—H10H	109.2	C207—C208—H20H	109.0
C109—C108—H10H	109.2	C209—C208—H20H	109.0
H10G—C108—H10H	107.9	H20G—C208—H20H	107.8
C108—C109—C110	110.3 (2)	C208—C209—C210	109.8 (2)
C108—C109—H10E	109.6	C208—C209—H20C	109.7
C110—C109—H10E	109.6	C210—C209—H20C	109.7
C108—C109—H10F	109.6	C208—C209—H20D	109.7
C110—C109—H10F	109.6	C210—C209—H20D	109.7
H10E—C109—H10F	108.1	H20C—C209—H20D	108.2
C109—C110—C101	115.04 (18)	C209—C210—C205	109.65 (19)
C109—C110—C105	109.55 (17)	C209—C210—C201	115.13 (18)
C101—C110—C105	117.18 (17)	C205—C210—C201	117.16 (17)
C109—C110—H110	104.5	C209—C210—H210	104.4
C101—C110—H110	104.5	C205—C210—H210	104.4
C105—C110—H110	104.5	C201—C210—H210	104.4
C101—C111—H11E	109.5	C201—C211—H21H	109.5
C101—C111—H11F	109.5	C201—C211—H21I	109.5
H11E—C111—H11F	109.5	H21H—C211—H21I	109.5
C101—C111—H11G	109.5	C201—C211—H21J	109.5
H11E—C111—H11G	109.5	H21H—C211—H21J	109.5
H11F—C111—H11G	109.5	H21I—C211—H21J	109.5
C102—C112—H11N	109.5	C202—C212—H21Q	109.5
C102—C112—H11O	109.5	C202—C212—H21R	109.5
H11N—C112—H11O	109.5	H21Q—C212—H21R	109.5
C102—C112—H11P	109.5	C202—C212—H21S	109.5
H11N—C112—H11P	109.5	H21Q—C212—H21S	109.5
H11O—C112—H11P	109.5	H21R—C212—H21S	109.5
C105—C113—H11H	109.5	C205—C213—H21E	109.5
C105—C113—H11I	109.5	C205—C213—H21F	109.5
H11H—C113—H11I	109.5	H21E—C213—H21F	109.5
C105—C113—H11J	109.5	C205—C213—H21G	109.5
H11H—C113—H11J	109.5	H21E—C213—H21G	109.5
H11I—C113—H11J	109.5	H21F—C213—H21G	109.5
C106—C114—H11Q	109.5	C206—C214—H21N	109.5
C106—C114—H11R	109.5	C206—C214—H21O	109.5
H11Q—C114—H11R	109.5	H21N—C214—H21O	109.5
C106—C114—H11S	109.5	C206—C214—H21P	109.5
H11Q—C114—H11S	109.5	H21N—C214—H21P	109.5
H11R—C114—H11S	109.5	H21O—C214—H21P	109.5
C116—C115—C101	116.98 (19)	C216—C215—C201	119.5 (2)
C116—C115—H11A	108.1	C216—C215—H21A	107.5
C101—C115—H11A	108.1	C201—C215—H21A	107.5
C116—C115—H11B	108.1	C216—C215—H21B	107.5
C101—C115—H11B	108.1	C201—C215—H21B	107.5
H11A—C115—H11B	107.3	H21A—C215—H21B	107.0
C117—C116—C115	113.5 (2)	C217—C216—C215	111.6 (2)
C117—C116—H11C	108.9	C217—C216—H21C	109.3
C115—C116—H11C	108.9	C215—C216—H21C	109.3
C117—C116—H11D	108.9	C217—C216—H21D	109.3
C115—C116—H11D	108.9	C215—C216—H21D	109.3
H11C—C116—H11D	107.7	H21C—C216—H21D	108.0
C119—C117—C118	125.1 (2)	C219—C217—C218	124.4 (2)
C119—C117—C116	119.3 (3)	C219—C217—C216	119.6 (2)
C118—C117—C116	115.6 (3)	C218—C217—C216	115.9 (2)
C117—C118—H11K	109.5	C217—C218—H21K	109.5
C117—C118—H11L	109.5	C217—C218—H21L	109.5
H11K—C118—H11L	109.5	H21K—C218—H21L	109.5
C117—C118—H11M	109.5	C217—C218—H21M	109.5
H11K—C118—H11M	109.5	H21K—C218—H21M	109.5
H11L—C118—H11M	109.5	H21L—C218—H21M	109.5
C117—C119—C120	129.7 (2)	C217—C219—C220	130.1 (3)
C117—C119—H119	115.1	C217—C219—H219	115.0
C120—C119—H119	115.1	C220—C219—H219	115.0
O102—C120—O101	121.8 (2)	O202—C220—O201	122.0 (2)
O102—C120—C119	123.6 (3)	O202—C220—C219	117.8 (2)
O101—C120—C119	114.6 (2)	O201—C220—C219	120.1 (2)
			
C111—C101—C102—C103	−75.5 (3)	C211—C201—C202—C203	−73.9 (3)
C115—C101—C102—C103	167.9 (2)	C215—C201—C202—C203	170.1 (2)
C110—C101—C102—C103	48.7 (3)	C210—C201—C202—C203	51.0 (3)
C111—C101—C102—C112	50.5 (3)	C211—C201—C202—C212	52.9 (3)
C115—C101—C102—C112	−66.1 (3)	C215—C201—C202—C212	−63.1 (3)
C110—C101—C102—C112	174.7 (2)	C210—C201—C202—C212	177.8 (2)
C112—C102—C103—C104	175.9 (2)	C212—C202—C203—C204	175.3 (3)
C101—C102—C103—C104	−55.6 (3)	C201—C202—C203—C204	−56.3 (3)
C102—C103—C104—C105	59.0 (3)	C202—C203—C204—C205	57.8 (3)
C103—C104—C105—C106	−172.56 (19)	C203—C204—C205—C206	−170.7 (2)
C103—C104—C105—C113	70.1 (2)	C203—C204—C205—C213	72.6 (3)
C103—C104—C105—C110	−55.0 (2)	C203—C204—C205—C210	−53.0 (3)
C113—C105—C106—C107	−100.2 (3)	C204—C205—C206—C207	141.0 (2)
C104—C105—C106—C107	141.4 (3)	C213—C205—C206—C207	−100.2 (3)
C110—C105—C106—C107	24.1 (3)	C210—C205—C206—C207	24.0 (3)
C113—C105—C106—C114	76.4 (3)	C204—C205—C206—C214	−40.7 (3)
C104—C105—C106—C114	−42.0 (3)	C213—C205—C206—C214	78.1 (3)
C110—C105—C106—C114	−159.3 (2)	C210—C205—C206—C214	−157.8 (2)
C114—C106—C107—C108	−178.1 (3)	C214—C206—C207—C208	−178.7 (3)
C105—C106—C107—C108	−1.5 (4)	C205—C206—C207—C208	−0.4 (4)
C106—C107—C108—C109	9.1 (4)	C206—C207—C208—C209	7.9 (4)
C107—C108—C109—C110	−40.1 (3)	C207—C208—C209—C210	−39.0 (3)
C108—C109—C110—C101	−161.0 (2)	C208—C209—C210—C205	64.0 (3)
C108—C109—C110—C105	64.5 (3)	C208—C209—C210—C201	−161.3 (2)
C102—C101—C110—C109	179.40 (19)	C206—C205—C210—C209	−54.8 (2)
C111—C101—C110—C109	−56.4 (3)	C204—C205—C210—C209	−173.93 (19)
C115—C101—C110—C109	60.2 (2)	C213—C205—C210—C209	63.9 (3)
C102—C101—C110—C105	−49.7 (2)	C206—C205—C210—C201	171.57 (18)
C111—C101—C110—C105	74.5 (2)	C204—C205—C210—C201	52.4 (3)
C115—C101—C110—C105	−168.90 (19)	C213—C205—C210—C201	−69.7 (3)
C106—C105—C110—C109	−54.5 (2)	C211—C201—C210—C209	−58.6 (3)
C113—C105—C110—C109	64.8 (2)	C202—C201—C210—C209	176.90 (19)
C104—C105—C110—C109	−174.01 (18)	C215—C201—C210—C209	58.0 (2)
C106—C105—C110—C101	172.11 (18)	C211—C201—C210—C205	72.6 (2)
C113—C105—C110—C101	−68.6 (2)	C202—C201—C210—C205	−52.0 (2)
C104—C105—C110—C101	52.6 (2)	C215—C201—C210—C205	−170.88 (19)
C102—C101—C115—C116	−56.7 (3)	C211—C201—C215—C216	−178.0 (2)
C111—C101—C115—C116	−177.1 (2)	C202—C201—C215—C216	−57.6 (3)
C110—C101—C115—C116	62.3 (3)	C210—C201—C215—C216	60.8 (3)
C101—C115—C116—C117	−171.6 (2)	C201—C215—C216—C217	−171.8 (2)
C115—C116—C117—C119	116.5 (3)	C215—C216—C217—C219	109.5 (3)
C115—C116—C117—C118	−64.2 (4)	C215—C216—C217—C218	−68.7 (3)
C118—C117—C119—C120	−2.4 (5)	C218—C217—C219—C220	3.3 (5)
C116—C117—C119—C120	176.8 (3)	C216—C217—C219—C220	−174.7 (3)
C117—C119—C120—O102	2.9 (5)	C217—C219—C220—O202	178.5 (3)
C117—C119—C120—O101	−175.8 (3)	C217—C219—C220—O201	−0.6 (5)

### Hydrogen-bond geometry (Å, °)

**Table:**

*D*—H···*A*	*D*—H	H···*A*	*D*···*A*	*D*—H···*A*
O101—H101···O202	0.82	1.82	2.625 (3)	168
O201—H201···O102	0.82	1.90	2.700 (2)	164
C212—H21R···O202^i^	0.96	2.60	3.519 (4)	159

Symmetry codes:  (i) −*x*+5/2, −*y*+2, *z*+1/2.
